# Editorial: Footprints of immune cells in the type 1 diabetic pancreas, volume II

**DOI:** 10.3389/fendo.2024.1367245

**Published:** 2024-02-06

**Authors:** Gustaf Christoffersson, Georgia Fousteri

**Affiliations:** ^1^ Department of Medical Cell Biology, SciLifeLab, Uppsala University, Uppsala, Sweden; ^2^ Division of Immunology, Transplantation, and Infectious Diseases, Diabetes Research Institute, Istituti di Ricovero e Cura a Carattere Scientifico (IRCCS) San Raffaele Hospital, Milan, Italy

**Keywords:** type 1 diabetes, immune cells, autoimmunity, pancreas, immunology

Investigations of immune cells have been central in the search for understanding the causes and mechanisms of type 1 diabetes (T1D), a disorder caused by an autoimmune reaction against the insulin-producing beta cells of the pancreas. In this second volume of the Research Topic “*Footprints of Immune Cells in the Type 1 Diabetic Pancreas*”, we have again invited researchers to submit papers on how immune cells and their products leave marks on the T1D pancreas.

It has become increasingly clear that assessing various parameters of the pancreas is important in T1D research for a wide variety of reasons. Immune cells in circulating blood, and other more peripheral measurements do not necessarily correlate well with the conditions in the pancreas. With the increased availability of high quality donated human pancreata (for example through the nPOD program), increasing quality and amount of clinical imaging data [e.g. MRI (1), PET (2)], and different spatial omics approaches (3), we have learnt that in conjunction to assessing e.g autoreactive immune cells, we need also to account for the different changes that occur in the organ itself, such as with exocrine tissue (4), vasculature (5), nerves (6), and resident immune cells (7).

One such feature relating to the beta cell itself is the enigmatic hyperexpression of HLA-I seen in human pancreata from T1D donors. In a review by Russell et al., the authors discuss the evidence for beta cell-released interferons as main drivers of this upregulation. Plausible reasons for increased amounts of interferons in islets are brought up, such as an antiviral response, and the infiltration of autoreactive immune cells. STAT signalling likely plays a role in this, and increased STAT1 expression has been observed in T1D islets, and correlated with HLA-I expression. The recent conclusion of the BANDIT clinical trial brings encouraging insights. Baricitinib, a Janus kinase (JAK) inhibitor renowned for its impact on autoimmune diseases, demonstrated promising potential in preserving β-cell function in individuals with recent-onset T1D (8). Thus, in line with studies in NOD mice (9, 10), inhibition of JAK-STAT signalling upstream may be a promising target in preventing T1D in humans.

The role of peripheral neurons and their interplay with immune cells is a growing field of study within immunology research, and data from both mice and humans point to important connections between the two systems also in T1D. Corral-Pujol et al. took a different approach to the study of the nervous system and T1D in NOD mice. They found signs of neurodegenerative processes in dorsal root ganglia, both histologically and transcriptionally. These observations, possibly pointing to alterations in sensory neurons during T1D onset in this preclinical model, may partly explain susceptibility to disease and revealed an extra-pancreatic site where neuronal changes take place adding up new knowledge to in the first observations on sensory neuron involvement in T1D (11).

In a paper by Šoić et al., the authors take an approach to look into a potential peripheral biomarker of T1D progression. The complement system and the complement component C3 are key factors in inflammatory conditions, including T1D, where elevated circulating levels are seen in individuals with the disease. As glycosylation of proteins increases with increasing glycemia, this study looked at the amount of N-glycosylated complement component C3 as a naturally compounded biomarker of T1D. In an assessment of C3 N-glycans and T1D complications, the authors found that the biomarker did not associate with secondary complication severity nor predicted it, but future prospective studies may provide interesting insights into how this biomarker could be used for early diagnosis.

Bruggeman et al. took a detailed and time-resolved approach to investigate trafficking of immune cells to the pancreas of NOD mouse. By assessing the pancreas, the pancreatic draining lymph nodes, and peripheral blood every 1-2 weeks during disease development in this mouse model of T1D, they charted a more complete picture of the dynamics of immune cell infiltration and phenotypes. As has been previously found, the immune cell signature of peripheral blood did not always correlate with what was present in the pancreas or the pancreatic draining lymph nodes. They found an early influx of neutrophils up to eight weeks of age in the mice, while dendritic cells seemed to move in and out of the pancreas to and from the lymph nodes. CD4^+^ and CD8^+^ T cells in the pancreas were mostly of in a naïve state up to week 12, when a shift into an effector memory phenotype was observed. T cells in peripheral blood and pancreatic draining lymph nodes remained largely naïve, however in blood, there was an emergence of pre-effector-like T cells that probably are the cells that enter the pancreas. This study provided new insights into the dynamics of immune cells trafficking in the NOD mouse, and stipulated a detailed chart, including transcriptomics, for further understanding of the important steps leading to the onset of T1D.

Overall, this Research Topic shows the broad approach that needs to be taken in order to both fully understand the natural history of disease in T1D, and to explore potential new therapeutics and biomarkers for early detection, disease monitoring, and treatment. We have made significant progress during the last years in understanding some of the processes in the pancreas leading up to disease. Now, the emergence of new techniques for the study of immune cells in this organ will hopefully take us even further, and help us see even their footprints.

## Author contributions

GC: Writing – original draft, Writing – review & editing. GF: Writing – original draft, Writing – review & editing.
